# *Enterococcus faecalis* Subverts and Invades the Host Urothelium in Patients with Chronic Urinary Tract Infection

**DOI:** 10.1371/journal.pone.0083637

**Published:** 2013-12-10

**Authors:** Harry Horsley, James Malone-Lee, David Holland, Madeleine Tuz, Andrew Hibbert, Michael Kelsey, Anthony Kupelian, Jennifer L. Rohn

**Affiliations:** 1 Centre for Clinical Science and Technology, Research Department of Clinical Physiology, Division of Medicine, University College London, London, United Kingdom; 2 Imaging Suite, Royal Veterinary College, London, United Kingdom; 3 Department of Microbiology, The Whittington Hospital NHS Trust, London, United Kingdom; University Medical Center Utrecht, Netherlands

## Abstract

Bacterial urinary tract infections (UTI) are a major growing concern worldwide. Uropathogenic *Escherichia coli* has been shown to invade the urothelium during acute UTI in mice and humans, forming intracellular reservoirs that can evade antibiotics and the immune response, allowing recurrence at a later date. Other bacterial species, such as *Staphylococcus saprophyticus*, *Klebsiella pneumonia* and *Salmonella enterica* have also been shown to be invasive in acute UTI. However, the role of intracellular infection in chronic UTI causing more subtle lower urinary tract symptoms (LUTS), a particular problem in the elderly population, is poorly understood. Moreover, the species of bacteria involved remains largely unknown. A previous study of a large cohort of non-acute LUTS patients found that *Enterococcus faecalis* was frequently found in urine specimens. *E. faecalis* accounts for a significant proportion of chronic bladder infections worldwide, although the invasive lifestyle of this uropathogen has yet to be reported. Here, we wanted to explore this question in more detail. We harvested urothelial cells shed in response to inflammation and, using advanced imaging techniques, inspected them for signs of bacterial pathology and invasion. We found strong evidence of intracellular *E. faecalis* harboured within urothelial cells shed from the bladder of LUTS patients. Furthermore, using a culture model system, these patient-isolated strains of *E. faecalis* were able to invade a transitional carcinoma cell line. In contrast, we found no evidence of cellular invasion by *E. coli* in the patient cells or the culture model system. Our data show that *E. faecalis* is highly competent to invade in this context; therefore, these results have implications for both the diagnosis and treatment of chronic LUTS.

## Introduction

Urinary tract infection (UTI) is a significant cause of morbidity, ranking as one of the most prevalent infectious diseases worldwide [1,2]. By the age of 24, nearly one third of women will have sought medical attention for an acute, self-limiting UTI and between 15-25% of this group will suffer from a recurrent or chronic form of this disease [2–5]. Acute UTI is not diagnostically challenging [6], as the rapid onset of urinary frequency and dysuria are clear indicators of the pathology.

Less clear cut are lower urinary tract symptoms (LUTS), a collective term describing a host of urological manifestations, including symptoms of urine storage and voiding, and pain attributed to the lower urinary tract [7]. While the role of infection in the generation of acute symptoms is well recognised, an infective aetiology in other LUTS is not typically assumed. In fact, most current guidance on the management of LUTS calls for the exclusion of UTI using routine urinalysis methods [8,9]. In this context, the term LUTS had become synonymous with non-infectious disease. The clinical features of UTI and LUTS show considerable overlap, however, and the prevalence of both disorders rises dramatically with age [10–13]. 

Our research centre and others have found that the tests deployed to screen for UTI are largely inadequate, particularly in patients who do not present with classic acute infective symptoms [14–16]. Although LUTS can undoubtedly be caused by other factors (e.g. carcinoma, urethral stricture, prostatic disease, bladder stones or affective disorders such as those common in multiple sclerosis [17,18]), we now know that patients scoring as negative on routine tests for infection might in fact harbour a low-grade bacterial pathology [19]. 

By far the most prevalent bacterial species implicated in acute UTI is *E. coli*, which is responsible for as many as to 90% of diagnosed cases of nosocomial and community-acquired bladder infection [20]. Murine models of acute UTI have shown that uropathogenic *E. coli* (UPEC) invades and forms intracellular bacterial communities (IBCs) in the bladder where it is able to evade immune surveillance and a number of systemic antibiotic treatments [5,21–27]. The findings from these studies have resulted in a well-accepted model of the acute UTI UPEC life cycle [25]. Adhesion and invasion into the host cell cytoplasm are closely followed by three distinct stages of the intracellular bacterial community (IBC) lifecycle. During early IBC, loose collections of bacillus bacteria rapidly divide inside the cytoplasm proper. In middle IBC, daughter cells exhibiting a coccoid morphology pack tightly producing a biofilm-like pod [23]. At the late IBC stage, bacteria at the periphery of the intracellular biofilms regain a rod morphology and become highly motile, leading to bacterial efflux and re-infection of adjacent cells [25]. 

Infected umbrella cells will be shed from the epithelial lining into the urine. Such sloughing is known in both mice and humans to be a common response to infection [24,28–31]. This dramatic cell shedding response leaves a gap in the epithelial layer, exposing naive transitional cells (proximal to the submucosal coat) to *de novo* UPEC invasion, a process which has been proposed to create quiescent intracellular reservoirs (QIR) responsible for latent recurrent and low-level chronic infection in mice [5,22,26,31,32]. Although QIR have not been directly observed in human patients, there is much evidence to suggest their existence [5,22,26,31,33–35]. Other IBC stages above, namely bacterial filamentation, have also been described in acute human UTI [24], although murine models remain more thoroughly studied.

Given the prevalence of UPEC as a causative agent in UTI, UPEC remains the most widely studied uropathogen. It is now recognized, however, that urothelial invasion may not be restricted to *E. coli* alone, with *Staphylococcus saprophyticus* [36] and *Klebsiella pneumonia* [37] also exhibiting UPEC-like intracellular lifestyles in experimental murine acute UTI. Recently, we explored an infective aetiology in LUTS using a traditional gentamicin protection assay, in which shielded bacteria were enumerated after extracellular bacteria were killed off by antibiotics [19]. In this study, *Enterococcus faecalis*, *Streptococcus anginosus, E. coli*, and *Proteus mirabilis* were shown to be closely associated with the shed cells. Although this assay is a trusted and well-tested method for detecting intracellular bacteria [38,39], the information gleaned from this technique is indirect. Furthermore, antibiotic-susceptible *E. faecalis* readily forms heavily antibiotic-resistant biofilms which could be responsible for false-positive outputs [40,41]. 

In the LUTS study described above, *E. faecalis* was the most cell-associated pathogen described, much more so than *E. coli*. In addition, *E. faecalis* is frequently isolated in acute UTI [10,24] and non-dysuric LUTS patients [19], and is commonly implicated in catheter-associated and chronic urinary tract infection [42,43]. In the past few years *Enterococcus spp.* have received a significant amount of attention. This opportunistic uropathogen is of particular concern in the clinical setting, where multi-drug resistant strains are frequently involved in hospital-acquired infection. Moreover, the rapid acquisition of antibiotic resistance, biofilm formation [40,41] and the innate ability to thrive and persist in the urinary tract make UTIs caused by *Enterococcus* spp. particularly difficult to eradicate [42,44–47]. Despite its commonness in bladder infections, aside from our recent paper [19], there have been no reports of *E. faecalis* associated with patient urothelial cells, nor any description of its potential intracellular invasion more direct than the antibiotic protection assay. We therefore set out to explore the role of cell shedding and bacterial invasion in LUTS in more detail.

## Results

### Significant urothelial shedding in the LUTS bladder

In order to study the role of infection and inflammation in LUTS, and to determine whether shed epithelial cells could be used as a further means of studying intracellular infection in more detail, we recruited 705 patients presenting at first visit to the clinic, 606 females and 99 males with a mean age of 51 years (sd=17.5). Of the 705 urine samples, 522 (74%) were found to be negative on routine mid-stream urine (MSU) culture (using a culture positive threshold of ≥ 10^5^ cfu ml^-1^ of a single known uropathogen) and 183 (26%) were positive. It should be noted that this routine MSU culture threshold, while standard for the UK and some other countries, misses significant infection in our clinical experience. For main demographic data and symptomatology please see Figure **S1**. 

Pyuria (white blood cells [WBC] µl^-1^), quantified immediately in fresh, unspun specimens of urine, is the best clinical indicator of UTI currently available. The patients were categorised into three groups according to pyuria expression: (1) for zero pyuria; (2) for pyuria 1 to 9 WBC µl^-1^; and (3) for pyuria ≥10 WBC µl^-1^. Pyuria ≥10 WBC µl^-1^ has long been advocated as a diagnostic threshold to discriminate between the presence or absence of lower urinary tract pathology. First described by Dukes in 1928 [48], this diagnostic threshold does not withstand contemporary scientific scrutiny [16], and there are no published data demonstrating that pyuria of 1 to 9 WBC µl^-1^ is non-pathological [49]. Indeed, 1-9 WBC µl^-1^ does predict underlying disease [49], so we thought it prudent to include this category. 

**Figure 1:**
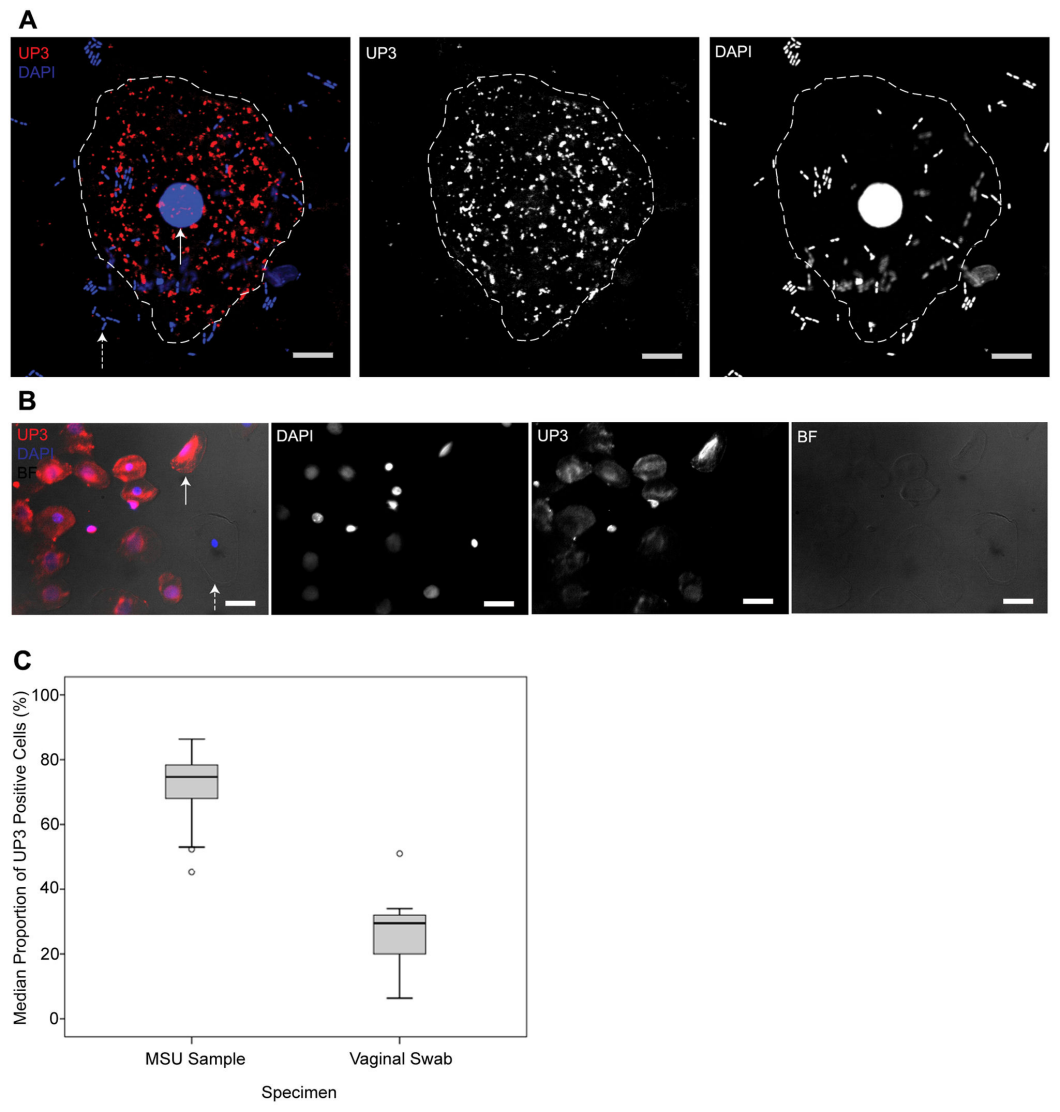
Uroplakin-3 (UP3) immunofluorescence. (A) Confocal image of a UP3-positive urothelial cell shed into the urine of a LUTS patient, a composite showing the expression of UP3 in red and DAPI-stained host (solid white arrow) and bacterial (broken white arrow) DNA in blue. Each channel is also shown in monochrome. The boundary of the cell is represented by a broken white line. White scale bar is 10µm. (B) Epi-fluorescent image of UP3-positive urothelial cells (solid white arrow) and a single UP3-negative epithelial cell (broken white arrow) harvested from the urine of a patient with LUTS. The composite image shows UP3 in red, host DNA in blue and the brightfield (BF) channel in greyscale. Channels are also shown separately in monochrome. White scale bar is 50µm. (C) Graph showing the median proportion of UP3-positive cells found in MSU samples (N=22) when compared with vaginal swabs (N=22). 75% (Q1=68, Q3=78.5) of the cells found in the MSU samples were UP3-positive in contrast to only 25% (Q1=19, Q3=32) in the vaginal swabs (U=1, p <.001).

In addition to WBC, mouse and human bladders shed urothelial cells into the urine as part of an innate immune response to bacterial insult in acute UTI [24,28–31]. Therefore, we also counted epithelial cells (EPC µl^-1^) in the urine of LUTS patients. First, we recognized the requirement for proof of cellular origin as a proportion of these cells could be contaminants originating from the genitalia and perineum. Uroplakin-III (UP3) is expressed solely on the asymmetric unit membrane of urothelial cells [50,51]. Therefore, we targeted this glycoprotein using immunofluorescence to determine the proportion of urinary epithelial cells that originated in the urinary tract (Figure **1A, B**). A subset of 44 randomly selected LUTS patients was included in this experiment. MSU samples from 22 patients with chronic LUTS (F=22; mean age=51; sd=19) were compared with vaginal swabs from 22 further chronic LUTS patients (F=22; mean age=50; sd=20). The median percentage of UP3-positive cells in the MSU samples was 75% (Q1=68, Q3=78.5) but only 25% (Q1=19, Q3=32) in the vaginal swabs (Figure **1C**). The results of a Mann-Whitney test show the proportion of UP3-positive cells found in the urine of chronic LUTS patients to be significantly higher than that in the vagina (U=1, p <.001). Therefore we could be confident that our cell analyses were representative of underlying pathology of the bladder. 

**Figure 2:**
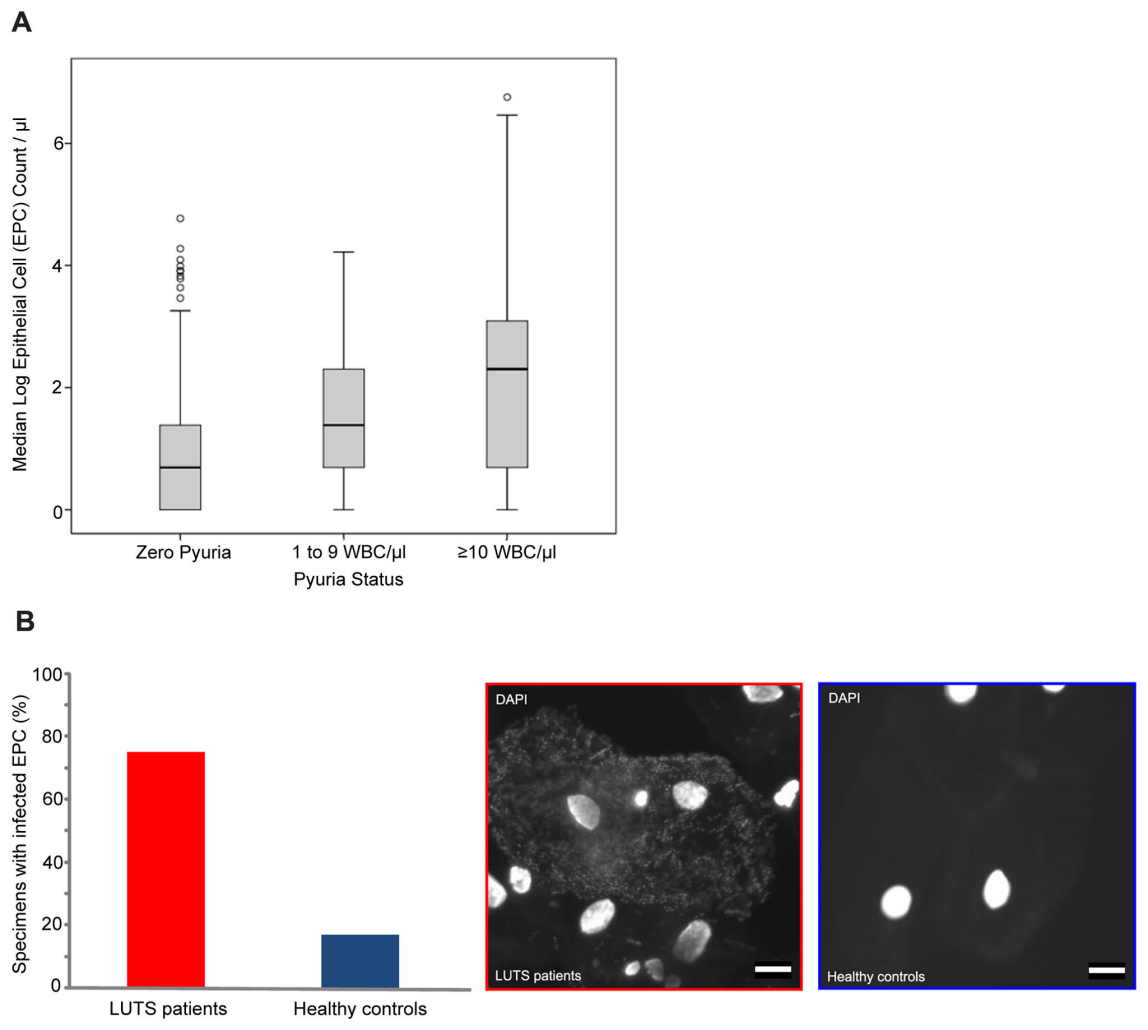
Urinary epithelial cell counts and bacterial pathology. (A) Relationship between the median log urinary epithelial count (EPC=epithelial cell) and the level of pyuria observed in the urine of LUTS patients (WBC=white blood cell). The mean log epithelial cell counts at each state of pyuria proved to be significantly different. (B) Bar chart showing the percentage of specimens with bacteria-association urinary epithelial cells (EPC) in LUTS patients (75%) and healthy controls (17%). Representative images of infected urinary EPC from LUTS patients (red) and normal EPC for healthy controls (blue) shown for reference. White scale bar is 10µm.

We counted shed EPC in the 705 patient cohort (Figure **2A**). The EPC counts were positively skewed and log transformation had a limited normalisation effect, changing the skewness from 13.4 to 0.63. We therefore compared the median log_10_ epithelial cell count (log_10_ EPC µl^-1^) between categories, non-parametrically. Figure **2A** shows clear between group differences between these categories (Kruskal-Wallis Χ^2^ = 75, p<.001, df=2). Post hoc analysis using Mann-Whitney test comparisons with a Bonferroni correction confirmed that all three groups differed among one another (p<.001). A similar comparison between the 522 (74%) showing a negative MSU culture and the 183 (26%) that were positive showed no between group differences (U=19x10^3^, P=.5). Therefore, the degree of EPC shedding corresponds to amount of pyuria, and by association, severity of infection.

In summary, this evidence supports the murine model of bacteria-induced urothelial inflammation and shedding in LUTS patients. Given that infection seemed to correlate with symptoms in this cohort, we set out to study the underlying microbiology in closer detail. 

### Implicating intracellular *E. faecalis* bacterial infection in the aetiopathology of LUTS

As with previous work exploring cellular invasion during acute UTI in humans [24], we inspected shed urothelial cells from LUTS patients with non-acute UTI for signs of bacterial association and intracellular pathology. Prior to imaging, the urine samples were cultured on chromogenic agar and any arising bacteria identified using a rigorous series of biochemical assays as outlined in the methods section. 

For this experiment we studied a randomly selected subset of 48 specimens using epi-fluorescent microscopy to identify bacterial involvement. MSU samples were donated by 24 female LUTS patients (mean age=52; *sd*=10.7) and compared with an equal number of samples from female healthy normal controls (*N*=24; mean age=48; *sd*=9.9). Although these 24 LUTS patients were negative for routine MSU culture, growing <10^5^ cfu ml^-1^ of a single known uropathogen, 10 exhibited growth of *E. coli* and/or *E. faecalis* with the addition of other species. One of these samples exhibited growth of only *E. coli* and *E. faecalis* with no other species present which gave us the opportunity to compare how each bacterium behaved. This sample was selected for confocal microscopy to explore intracellular pathology. 

Our previous work, which explored intracellular colonisation of the LUTS bladder, presented data from antibiotic protection assays alone [19]. This microbiological data is indirect and can be misleading, as detergents may liberate live membrane-bound bacteria. This technique is hindered further by the presence of extracellular biofilms which may be unaffected by even high concentrations of antibiotics. Therefore, to decisively inspect these cells for intracellular colonisation we conducted confocal laser-scanning microscopy in conjunction with a bank of extensive 3D digital analyses. 

**Figure 3:**
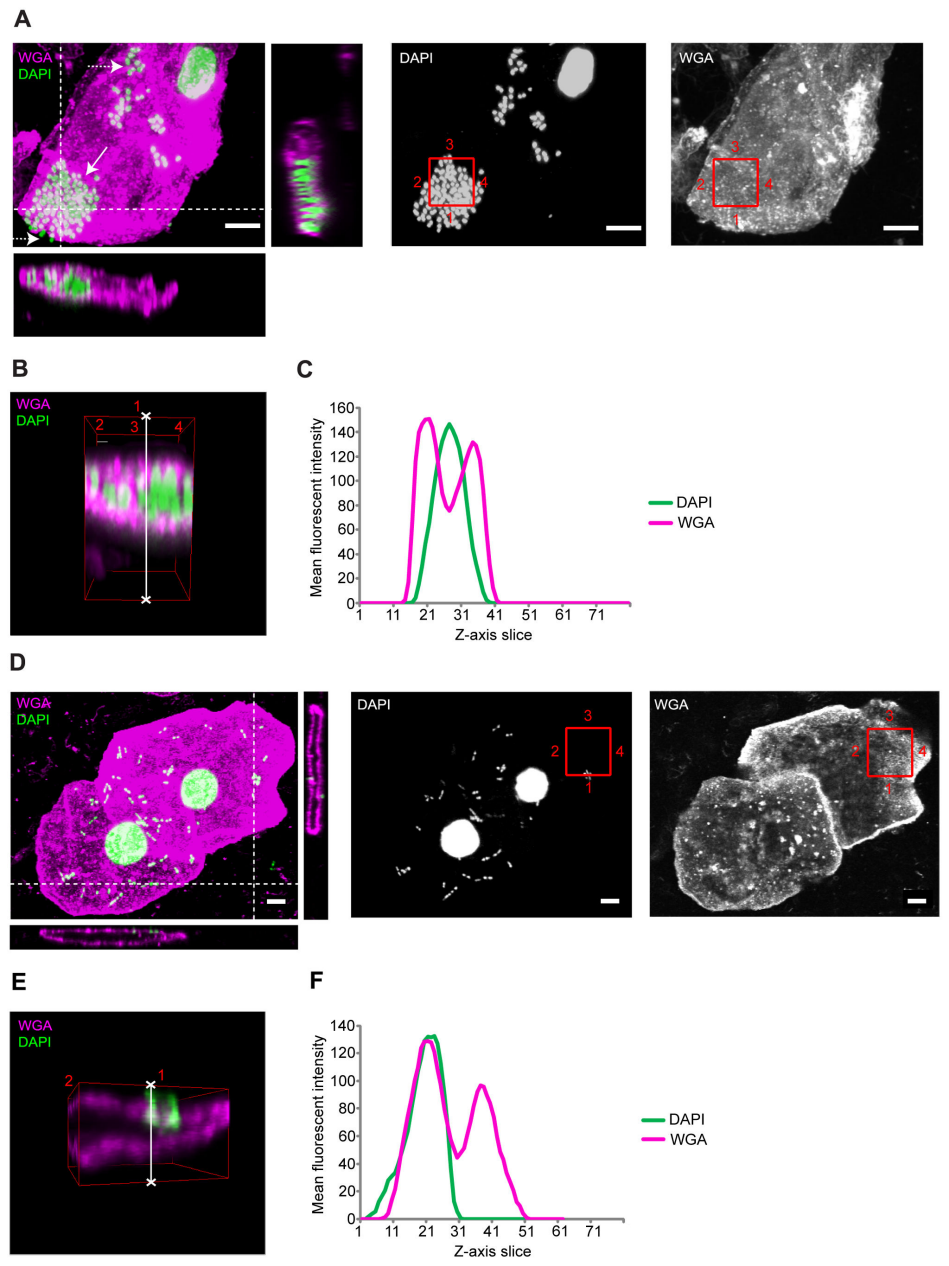
Bacterial invasion in shed urothelial cells. Prior to imaging, the urine sample was cultured on chromogenic agar and bacterial identities confirmed using a bank of biochemical assays as outlined in the methods section. By supporting the confocal data with culture results and bacterial identity we were able recognize bacteria by morphology during confocal analysis. (A) Maximum projection confocal image of a cell shed from the bladder of a LUTS patient with a mixed sub-threshold infection with *E. faecalis* and *E. coli*. The left image is a composite showing the wheat germ agglutinin (WGA)-stained plasma membrane in magenta and DAPI stained host and bacterial DNA in green. Solid white arrow highlights a cluster of intracellular coccoid bacteria, broken white arrow highlights extracellular coccoid bacteria. Bacteria were identified as *E. faecalis* owing to morphology. Orthogonal views are through entire Z-stack at a position corresponding to white broken lines on the left image, showing intracellular colonisation. The centre and right image show the respective DAPI and WGA channels in monochrome. White scale bar is 5µm. (B) A 3-dimensional volume through the entire Z-stack at a position corresponding to the red squares in image A (numbered for orientation); bacteria are clearly residing within the cell. (C) A region of bacterial colonisation was selected (highlighted by the white line in 3D construct B) and used to produce a Z-axis profile plot, which presents the average pixel intensity of a given channel moving through the 80-slice Z-stack. This graphical representation shows further evidence of cellular invasion, with the peak mean pixel intensity of the DAPI channel (*E. faecalis*, green) corresponding with a striking reduction in the WGA (plasma membrane, magenta) channel at slice 29 (centre of the cell). (D) Maximum projection confocal image of a cell shed from the same patient as above with a mixed sub-threshold infection with *E. faecalis* and *E. coli*. Images were analysed and presented as above. Bacteria were identified as *E. coli* owing to bacillus morphology. Orthogonal views show the *E. coli* to be strongly associated with the plasma membrane but entirely extracellular. White scale bar is 5µm. (E) 3-dimensional volume corresponding to the red squares in image D (numbered for orientation); again, the *E. coli* were shown to be ubiquitously extracellular. (F) A region of bacterial colonisation was selected (highlighted by the white line in 3D construct E) to produce a Z-axis profile plot. This graphical representation shows further evidence of external cellular colonisation alone. The peak mean pixel intensity of the DAPI channel corresponded with that of the WGA stained apical plasma membrane channel at slice 21.

On epi-fluorescent analysis, 75% (N=18) of the LUTS patient samples showed evidence of infected urothelial cells in comparison to only 17% (N=4) in the control group (Figure **2B**). Data from the confocal analysis of a representative sample suggested that *E. faecalis* and *E. coli* employ distinct pathological strategies in these patients, with only *E. faecalis* exhibiting cellular invasion. Figure **3** shows two representative cells shed from the bladder of a LUTS patient with a mixed sub-threshold infection with *E. faecalis* and *E. coli*. Cells that were associated with adherent extracellular bacilli (*E. coli*) did not contain intracellular bacterial communities (Figure **3D**). However, in cells exhibiting adherent, extracellular coccoid *E. faecalis* (Figure **3A**, broken white arrow), we found compelling evidence of intracellular pathology (Figure **3A**, white arrow, B, C). Although uropathogenic *E. coli* have the unusual ability to transform from a rod to coccoid morphology within the intracellular niche within the centre of a tightly packed IBC [23,24], we saw isolated intracellular coccoid forms, too dispersed to be coccoid *E. coli*, suggesting that the organism was indeed *E. faecalis*.

### LUTS patient-isolated *E. coli* does not invade cells in a cell culture model system

So far, we have shown that the presence of LUTS is associated with shedding of the urothelium, which is likely activated by the presence of bacteria. The confocal data from analysis of shed patient cells suggested that *E. faecalis* employed an invasive lifestyle whereas *E. coli* was ubiquitously extracellular. 

Given the difficulty of identifying in advance patient cells that harboured *E. coli* and/or *E. faecalis* in the absence of other species, we decided to use defined infections in cell culture to explore this issue further. We designed a cell culture system to model the infection process using five strains of *E. coli* isolated from routine MSU-culture negative LUTS patients. We infected a T24 transitional bladder cell line before, as with the shed urothelial cells, fluorescently staining and imaging using high-resolution confocal microscopy. The invasive properties of these strains were analysed using a series of 3D digital analyses.

**Figure 4:**
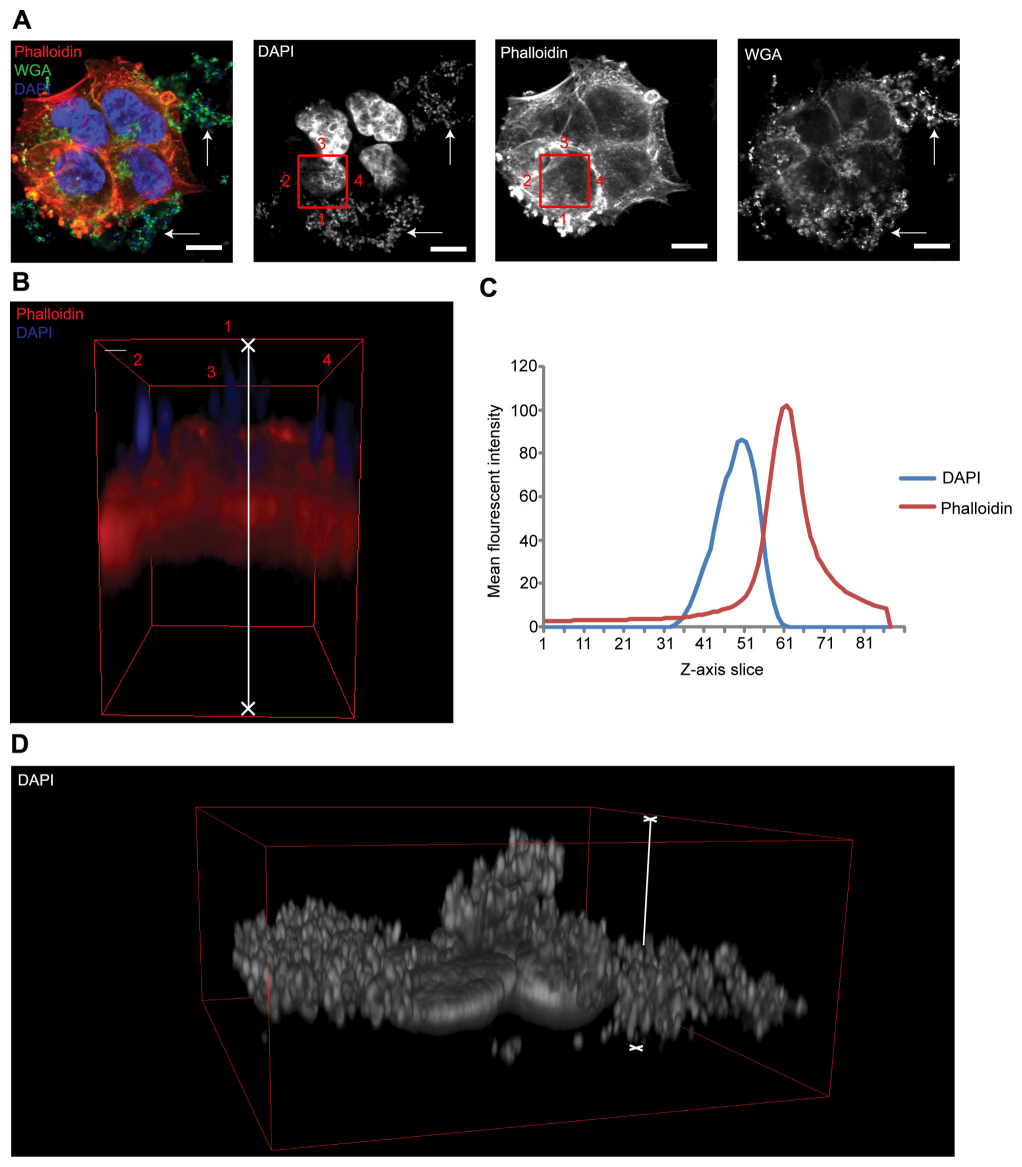
3D confocal analysis of cultured cells infected with LUTS-isolated *E. coli*. (A) Confocal image of a group of T24 cells infected for 2.5hrs with *E. coli* isolated from LUTS patients at an MOI of approximately 10-15 bacteria per mammalian cell. The left image is a composite showing the phalloidin-stained F-actin in red, wheat germ agglutinin (WGA) stained plasma membrane in green and DAPI stained host and bacterial DNA in blue. The left centre, right centre and far right images show the respective DAPI, phalloidin and WGA channels in monochrome. Solid white arrows highlight WGA-positive staining of an exopolymeric matrix secreted by *E. coli* during biofilm formation. White scale bar is 10µm. (B) A 3-dimensional volume through entire Z-stack at a position corresponding to the red squares in image A (numbered for orientation); the *E. coli* is clearly bound to the extracellular space. (C) A Z-axis profile plot was produced as in Figure 3. The DAPI channel is approximately 16 slices (in the Z-axis) from that of the phalloidin channel, confirming purely extracellular colonisation. (D) 3D construct of the cells shown in A with the DAPI channel shown alone. This image shows the extent of bacterial infection. Very deep and tightly packed *E. coli* biofilms can be seen covering the apical membrane of the T24 cells. The region of interest analysed in B and C is indicated with a white line.

All of the five *E. coli*-infected T24 cells exhibited marked bacterial adhesion and colonisation (See Figure **4A, D** for a representative example). Orthogonal views of Z-stack 3D constructs, however, showed the colonies of each strain of bacteria to be entirely extracellular (Figure **4B**). Further analysis using Z-axis profile plots supported these findings, with the peak mean pixel intensity of the DAPI labelled bacteria (blue) found to be some distance away (in the Z-axis) from that of the phalloidin labelled F-actin (red) (Figure **4C**). 

Remarkably, it appeared that these LUTS-isolated strains of *E. coli* formed tightly packed extracellular biofilms on the surface of the T24 cells (Figure **4D**). Positive staining with wheat germ agglutinin (WGA) in a gram-negative organism like *E. coli* signifies the secretion of an exopolymeric matrix, inherent in biofilm formation [52] [53] (Figure **4A**, white arrows). 

### LUTS patient-isolated *E. faecalis* invades cells in a cell culture model system

Confocal analysis of shed LUTS patient urothelial cells demonstrated cellular invasion by *E. faecalis*. To test further whether *E. faecalis* is competent to invade urothelial cells, as our earlier findings would suggest, we challenged the same T24 bladder cell culture model system as described above with five strains of *E. faecalis* isolated from routine MSU-culture negative LUTS patients. Again, the infected cells were imaged using confocal microscopy and explored extensively with 3D digital analyses.

**Figure 5:**
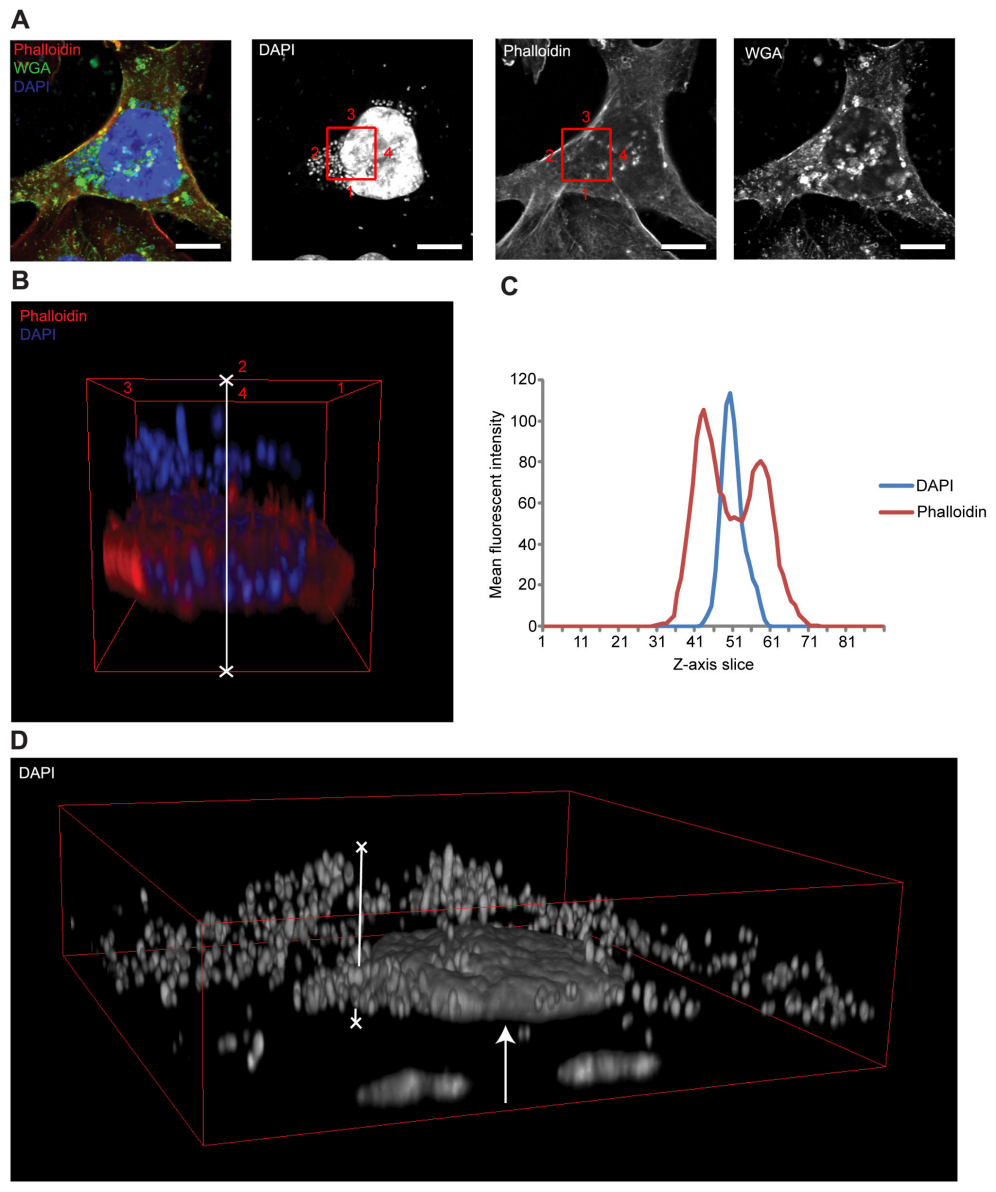
3D confocal analysis of cultured cells infected with LUTS-isolated *E. faecalis*. (A) Confocal image of a group of T24 cells infected for 2.5hrs with *E. faecalis* isolated from LUTS patients at an MOI of approximately 10-15 bacteria per mammalian cell. The left image is a composite showing the phalloidin stained F-actin in red, wheat germ agglutinin (WGA)-stained plasma membrane in green and DAPI stained host and bacterial DNA in blue. The left centre, right centre and far right images show the respective DAPI, phalloidin and WGA channels in monochrome. White scale bar is 10µm. (B) A 3-dimensional volume through entire Z-stack at a position corresponding to the red squares in image A (numbered for orientation). This construct shows clear and extensive invasion and colonisation of the T24 cell by *E. faecalis*. (C) A Z-axis profile plot was produced as in Figures 3 and 4. The peak mean pixel intensity of the DAPI channel corresponds with a striking reduction of the phalloidin channel at slice 51 (centre of the cell), confirming intracellular infection. (D) 3D construct of the entire cell as in Figure 5. This image shows the tightly packed intracellular biofilm like cluster of *E. faecalis* in close proximity to the T24 nucleus (white arrow).

Unlike *E. coli*, *E. faecalis* adhered to the T24 cells in looser, more diffuse clusters (See Figure **5** for a representative example). More organised colonies were evident, although they manifested close to the T24 nuclei and within the horizontal boundaries of the cells, which suggested cellular invasion (Figure **5A**). Z-stack 3D constructs of these bacterial colonies clearly showed all five of the LUTS patient isolated *E. faecalis* to be sandwiched within the intracellular space of the T24 cells (Figure **5B, D**). These findings were mirrored in the Z-axis profile plots, which showed a marked decrease in mean pixel intensity of phalloidin-labelled F-actin (red) and a striking increase in the mean pixel intensity of the DAPI labelled *E. faecalis* at mid-cell slices (Figure **5C**). 

Significant bacterial pathology associated with the urothelium is clearly evident in the LUTS population and the results of these 3D analyses support the hypothesis of intracellular colonisation by *E. faecalis* in LUTS patients. In contrast to the case of acute infection, it would appear that *E. coli* may not be ubiquitously invasive in the context of chronic low-grade UTI. It is important to note, however, that the lack of invasion may not necessarily preclude pathogenicity; the ability to form extracellular biofilms, as *E. coli* did in this study, could prove equally challenging to treat. 

## Discussion

Over the past decade, a model of the pathophysiology of UTI has solidified into accepted fact. Stemming from the initial elegant description of intracellular UPEC in mouse models of acute UTI [23], a number of studies have generally supported these initial findings. The data in mouse models is indeed robust [5,21–27]. It is worth noting, however, that the evidence for the presence of intracellular bacteria in human patients suffering acute UTI is confined to only one report of 80 women [24]. Another report has shown that UPEC isolated from women with acute cystitis were competent to form IBC in mice [27], but this evidence is not direct proof of their presence in the original patients. Hence, more data on the pervasiveness of intracellular bacteria in acute human UTI would be welcome.

In the case of non-acute, low-grade UTI responsible for chronic lower urinary tract symptoms, such as those exhibited by the patients in our clinic, even less is known about the bacterial lifecycle within the bladder. Our previous study showed that *E. coli* is not the sole or even foremost pathogen in these patients, and that a spectrum of species may be capable of causing such troublesome symptoms [19]. In that report we showed close association of four bacterial species – *E. faecalis, Streptococcus angiosus, E. coli and Proteus* *spp.* with epithelial cells shed from patients, suggestive of intracellular colonization, and further demonstrated that these bacterial isolates seemed competent to invade a bladder cell line, as assessed by an antibiotic protection assay. We were aware, however, that this assay, although widely trusted and used, could give misleading results if all extracellular bacteria were not killed by the antibiotics, for example if they were protected by biofilm formation or by very close association within niches in the external cell membrane. 

Our current efforts improve on these methods. We saw compelling evidence of intracellular bacteria in patient-isolated urothelial cells using fluorescence confocal microscopy. Furthermore, these data strongly suggested *E. faecalis* was the invasive pathogen whereas *E. coli* appeared to be associated with the surface membrane. Although UPEC at the centre of IBCs can exhibit a coccoid morphology, these biofilm-like pods in experimental mouse models are very tightly packed and UPEC residing at the circumference of this pod, along with extracellular adherent bacteria, maintain a more rod-shaped (or filamentous) morphology [5,23,25]. In contrast, in this study, the colonies observed were far more loosely organised and both extracellular and intracellular bacteria were equally coccoid in morphology. We concluded therefore, with corresponding culture results and phenotypic bacterial typing, that the invasive pathogen was indeed *E. faecalis*. Nevertheless, in future work, species-specific labelling using molecular methods such as PNA-FISH could be employed for definitive identification [54]. It will also be important to survey more patients to see how widespread this phenomenon is. 

When isolates of *E. coli* or *E. faecalis* were used to infect a human bladder cell line, which could be fixed post-infection and examined via confocal microscopy, we detected unequivocal intracellular colonization by five out of five isolates of *E. faecalis*. Surprisingly, although all five *E. coli* isolates demonstrated extensive extracellular adherence and biofilm formation, none were able to invade the cells. It is known that the bacterial virulence factor FimH is required for *E. coli* to invade bladder cells [55], so it is possible that our LUTS-derived isolates were deficient in some way for expression or sequence of this domain. However, this is unlikely, as we confirmed using a standard blood agglutination assay that our LUTS-derived *E. coli* expressed functional FimH (data not shown). Also, FimH is required for extracellular adhesion [56–58] and pathogenic biofilm formation [59], which all five strains were able to achieve in our tissue culture experiments. This suggests that *E. coli* isolated from these patients are indeed pathogenic but may lack the necessary downstream post-adhesion factors required for invasion. Further exploration of the FimH sequence in these strains and of other virulence factors could shed light on this interesting difference. 

Taken together, these results suggest that, in contrast to the case of acute UTI, *Enterococci* may be a key invasive pathogen in LUTS. The mechanism by which *E. faecalis* invades urothelial cells is unknown. However, it is likely that, as with *E. coli* invasion, a necessary first step would be adhesion, in which case various previously described adhesion proteins are likely to be involved [60]. Afterwards, it is possible that, like *E. coli*, *E. faecalis* may take advantage of the unique fusiform vesicle trafficking system used by urothelial cells to rapidly change the tissue surface area, and become passively engulfed [5]. Alternatively, it is known that *E. faecalis* are able to invade intestinal epithelial cells via the production of aggregation substance (AS) and therefore it is possible that a similar mechanism is involved in urothelial invasion [61]. Additional studies with more isolates will be needed to see how widespread *E. faecalis* is in LUTS patients, the invasive mechanisms involved and whether other species can also invade. Meanwhile, these data constitute, to our knowledge, the first report of definitive intracellular invasion of urothelial cells by *E. faecalis*. 

One of the problems of pinpointing an infective aetiology for LUTS lies in the poor sensitivity of routine urine testing, whose weakness has been amply demonstrated [14–16]. Infection associated with lower counts of bacteria, which may nevertheless be significant in the case of LUTS patients [19], tend not to be detected by urinary dipstick for nitrite or leukocyte esterase, nor by routine MSU cultures with high thresholds for what is considered “positive” (e.g. in the UK and many other countries, this breakpoint is ≥10^5^ cfu ml^-1^). Therefore, there is much interest in markers that might help diagnose such lower-grade urinary infections. Pyuria is one such validated marker. In addition, during acute UPEC-mediated UTI, inflammatory responses are known to cause urothelial cell shedding in both mice and humans [24,28–31]. This jettisoning is thought to be a defense mechanism to reduce bacterial burden in the urothelium. While some have dismissed urothelial cells in urine as contamination from the urogenital area during sample collection, our data show that the vast majority of these cells stain positive for uroplakin-3 and can therefore be considered to originate from the urothelium. In our study, epithelial shedding showed a strong relationship with pyuria: the higher the white blood cell count in the urine, the more epithelial cells were shed. Interestingly, even low pyuria counts (1 to 9 WBC µl^-1^) were associated with shedding, supporting the hypothesis that examining such cells in fresh urine, along with pyuria, might help diagnose infection undetected by dipstick or routine MSU culture. 

In mouse models, urothelial shedding has been proposed to facilitate the deeper invasion of the bladder by allowing access of bacteria to exposed transitional layers. Quiescent intracellular reservoirs (QIR) can result, leading to a chronic and/or recurrent infection situation [5,22,26,31,32]. Data from human recurrent UTI demonstrate that 68% of bacteriological recurrence is caused by identical bacterial strains to that of the index infection [33]. Although it could be argued that these relapses are caused by reintroduction of pathogen from faecal flora [3], same-strain infections can occur up to 3 years later [35] and the application of daily topical antibiotics to the perineum does not prevent recurrent episodes [34]. Although QIR have not been directly seen in human patients, these data are at least suggestive that QIR might play a role in intransigent LUTS. Further studies are needed to confirm this hypothesis.

In summary, our data suggest that some LUTS may be generated by low-grade intracellular infection of the bladder by *E. faecalis*. These results therefore may have far-reaching implications for our diagnosis, treatment and understanding of the aetiology of LUTS

## Materials and Methods

### Ethics statement

Ethical committee approval for human urine sampling was obtained from East Central London REC1 (Research Ethical Committee). All study participants gave written consent to participate in the study and the process was documented as per Good Clinical Practice (GCP) and MHRA guidelines. The participants were assigned randomly generated study numbers which were used to anonymise all data and samples. Analysis was carried out by blinded researchers. 

### Patient sampling

Sampling was conducted at Professor James Malone-Lee’s LUTS outpatient clinic, University College London, Division of Medicine. We sampled from 705 adults aged ≥18 years who were able to give consent and who were diagnosed with LUTS (see Figure **S1** for a summary of the cohort’s demographics and symptoms), excluding any patient with symptoms of acute UTI, with concurrent illnesses that in our opinion were likely to compromise the validity of the data, and pregnant women or those planning to conceive. When controls were required, we recruited with consent from staff that did not have any LUTS. All laboratory experiments used randomly selected sub-sets of the main patient cohort. Details can be found in the respective results sections. 

In all cases, subjects were provided with a sterile container and two hypoallergenic wipes. They were then given detailed (written and oral) instructions on meticulous MSU capture technique, namely (1) to wash their hands and thoroughly cleanse genital area with a hypoallergenic wipe to prevent contamination from the surrounding external genitalia; (2) to part labia or retract foreskin and urinate a small amount into the toilet before moving the container into the urine stream; and (3) to remove the container before urination was complete, thereafter to seal the container. The urine sample was divided into two aliquots. The first was submitted to immediate microscopy using a haemocytometer to enumerate leukocytes (WBC μl^-1^) and shed epithelial cells (EPC μl^-1^). The second aliquot, as per standard clinical guidelines, was sent for routine MSU culture at the Whittington Hospital NHS Trust, London, UK. The sampled urine was treated fresh, or after overnight storage at 4°C at the hospital laboratory. 1μl of urine was transferred by calibrated loop to chromogenic media, CPS3 (bioMerieux). The plate was incubated aerobically for 24 hrs at 37°C. Bacterial colonies were identified at the genus level by colour change. The result was reported as positive if greater than 10^5^ (MSU) colony forming units (cfu) ml^-1^ of a single known urinary pathogen were observed.

### Bacterial identification at the species level

To corroborate confocal data and identify bacteria prior to the invasion assays, the routine urine procedure above was repeated in-house with the addition of a dilution series and extensive phenotypic analyses to confirm bacterial identity at the species level. 

For each sample, 50μl of undiluted urine and three serial dilutions (1:10, 1:100 and 1:1000) were added to the respective quartile of a chromogenic CPS3 agar plate (bioMérieux) before aerobic incubation for 24 hours at 37°C. Following incubation, the different coloured colonies present on the chromogenic agar were identified using the manufacturer’s colour criteria. The colonies found in this study were identified as follows: *E. coli*; medium sized burgundy, pink or mauve colonies and *Enterococcus spp.*; small turquoise colonies.

To further confirm the identity as *E. coli*, the isolates were shown to be bacillus in morphology and negative under Gram staining, indole positive and identified as *E. coli* using API 20E (bioMérieux) biochemical test strips. Each of the *Enterococcus spp.* tested in this study were found to be *E. faecalis* owing to a coccoid morphology, positive Gram stain, negative catalase test, positive bile esculin test and by being identified as *E. faecalis* using API 20 Strep (bioMérieux) biochemical test strips. 

### Cell culture

A transformed cell line derived from a human bladder carcinoma (T24) [62] was kindly donated by Dr. Aled Clayton, Institute of Cancer and Genetics, School of Medicine, Cardiff University, United Kingdom. T24 cells were cultured at 37°C in a humidified incubator under 5% CO_2_ in 9 cm dishes in RPMI 1640 medium (Gibco) supplemented with 10% fetal bovine serum (FBS, PAA) and antibiotics (50 µg/ml penicillin and 50 mg/ml streptomycin; Gibco). Cells were maintained, pre-experimentation, by splitting 1:20 at 80% confluency. All work was carried out within sterile class-II flow cabinets under strict aseptic conditions. 

### Immunofluorescence staining of patient samples

80µl of a urine specimen was added to a Shandon single funnel cuvette assembly containing a pre-labeled glass slide and a Shandon filter card (Fisher Scientific). This assembly was centrifuged in a Shandon Cytospin 2 cytocentrifuge at 800rpm (≈75g rcf) for 5 minutes resulting in a visible disc of urinary particulate deposited on the slide, which was circumscribed with a hydrophobic barrier pen (ImmEdge pen, Vector Laboratories). The cells were fixed in 4% formaldehyde (Thermo Scientific, Fisher Scientific) in phosphate buffered saline (PBS, Sigma-Aldrich) at RT for 15 minutes. The formaldehyde was aspirated and the preparation washed three times with PBS at 5 minute intervals. For uroplakin-III staining, cells were permeabilised with 0.2% Triton-X100 (Sigma-Aldrich) in PBS for 5 minutes at RT followed by a single wash with PBS. The preparation was then blocked with 5% normal goat serum (Sigma-Aldrich) in PBS for 30 minutes prior to a 1 hour incubation with a 1:10 dilution of primary anti-uroplakin-III mouse monoclonal antibody (Progen Bioteknik) in 1% bovine serum albumin (BSA, Sigma-Aldrich). Following 3 further PBS washes, cells were incubated at RT for 40 minutes in a solution containing a 1:250 dilution of goat anti-mouse secondary antibody conjugated to Alexa Fluor-555 (Invitrogen), 1μg/μl of the DNA counterstain 4’’,6-diamidino-2-phenylindole, (DAPI, Sigma-Aldrich) and 1% BSA in PBS.

For assessing bacterial infection, after fixation as above, wheat germ agglutinin (WGA) conjugated to Alexa Fluor-488 (Invitrogen) was used to label the cell membrane to aid cellular identification and demarcation, and to assess biofilm formation where applicable. Following 15 minutes of incubation at RT with 1µg/ml WGA in Hank’s balanced salt solution minus phenol red (HBSS, Invitrogen), the labeling solution was removed and the cells washed twice at 5 minute intervals with HBSS. Fluorescent counterstaining of host and pathogen DNA was achieved through the addition of DAPI at 1µg/ml in PBS. After incubation for 15 minutes at RT, the DAPI solution was removed and the sediment washed twice in PBS. Following staining for uroplakin-III or infection, the preparations were immediately mounted with FluorSave reagent (Calbiochem) and a coverslip fixed in place with clear nail varnish. The percentage of UP3-positive cells was calculated by a blinded researcher using epi-fluorescent microscopy. Counts were carried out in triplicate. Images were also taken using scanning confocal microscopy on a Leica SP5. 

### Invasion assay

5 frozen *Enterococcus faecalis* and 5 frozen *Escherichia coli* strains previously isolated and typed from human LUTS patients were grown on fresh chromogenic CPS3 agar plates. After aerobic incubation for 24 hours at 37°C, a colony from each of these 2 bacterial cultures was transferred to a 5ml sterile aliquot of LB, and incubated aerobically at 37°C for 24 hours. Two of the 5 *E. coli* isolates underwent stationary incubation and the remaining 3 shaking incubation at 300rpm to control for FimH expression in these strains, as stationary growth has been reported to promote expression of this virulence factor [55]. In our hands, the two different methods of culture produced similar functional FimH behaviour. *E. faecalis* isolates were all incubated in a shaking incubator at 300rpm. The level of growth was checked for each bacteria using a spectrophotometer and diluted as necessary with fresh LB to an A600 of approximately 0.5 [63]. Lab-Tek II 8 well chamber slides (Nunc, Thermo Scientific) were coated with 200µl of FBS and incubated for 2 hours at 37°C before aspiration. T24 cells were plated at 8x10^4^ cells per well in a total volume of 400 µL and incubated for 24 hours at 37°C under 5% CO_2_ to allow cells to spread, then washed twice with PBS to remove routine antibiotics. Each of the bacterial LB cultures was diluted in CO_2_-independent media (CIM, Gibco) supplemented with 10% FBS (with routine antibiotics omitted) and 200µl added to the cultured T24 cells giving a multiplicity of infection (MOI) of approximately 10-15 bacteria per mammalian cell [63]. Cells were infected at 37°C for between 1 and 4 hours within a humidified aerobic incubator to optimise experimental parameters. 

Post infection, cells were inspected for morphological changes and viability before the addition of a combination of membrane-impermeable antibiotics to kill extracellular bacteria and to limit further cellular damage [19,38,39,63]. Briefly, infected CIM was carefully removed and a solution of gentamicin, linezolid and amoxycillin (Whittington Health NHS Trust Pharmacy, London) in 200µl fresh CIM was added at varying concentrations. Concentrations of gentamicin (200µg/ml), linezolid (25µg/ml) and amoxycillin (250µg/ml) were found to be most effective (adapted from [19]). The chamber slide was incubated, with antibiotics, aerobically for a further 24 hours at 37°C, after which the cells were fixed in 4% formaldehyde in PBS for 15 min at room temperature before washing three times in PBS at 5 minute intervals. The cell membranes were stained with 200µl WGA (1µg/ml) conjugated to Alexa Fluor-488 in HBSS minus phenol red for 15 min at RT. The labelling solution was removed and the cells washed twice at 5 minute intervals with HBSS. 0.2% Triton-X100 in PBS was then added and the cells allowed to incubate at room temperature for 5 min. Following removal of the Triton-X100, the permeabilised cells were washed once with PBS before staining with a 200µl solution of TRITC-conjugated phalloidin (0.6µg/ml)(Sigma-Aldrich), to label filamentous actin, and DAPI (1µg/ml) in PBS for 40 min at RT. The dual-labelling solution was gently aspirated and the cells washed 3 times in PBS before removal of the upper-well section of the Lab-Tek II chamber slide and immediate mounting as above. 

### Imaging and analysis

We performed epi-fluorescent microscopy on a Olympus CX-41 and Leica DM4000B upright microscope, and confocal laser scanning microscopy on a Leica SP5 microscope. Images were processed and analysed using Infinity Capture and Analyze V6.2.0, ImageJ 1.46r and the Leica Application Suite, Advanced Fluorescence 3.1.0 build 8587 Software.

## Supporting Information

Figure S1 **Demographics and symptoms.** (A) Key demographic information. (B) A four-way Venn diagram illustrating the overlap of symptoms amongst the 705 patients studied. The ellipses circumscribe patients who had one or more symptoms in the particular subset. Each ellipse corresponds to a numbered list of specific symptoms. The diagram is not scaled to the size of sets. Abbreviations; MSU (mid-stream urine culture); Pyuria (presence of white blood cells in the urine); WBC (white blood cell); inc (incontinence); LUTS (lower urinary tract symptoms); OAB (overactive bladder).
(TIF)
