# Supplementary figures and images for: Enterococcus faecalis Subverts and Invades the Host Urothelium in Patients with Chronic Urinary Tract Infection
Source: PLoS One. 2013 Dec 10;8(12):e83637. doi: 10.1371/journal.pone.0083637 (PMC3868479; doi:10.1371/journal.pone.0083637)

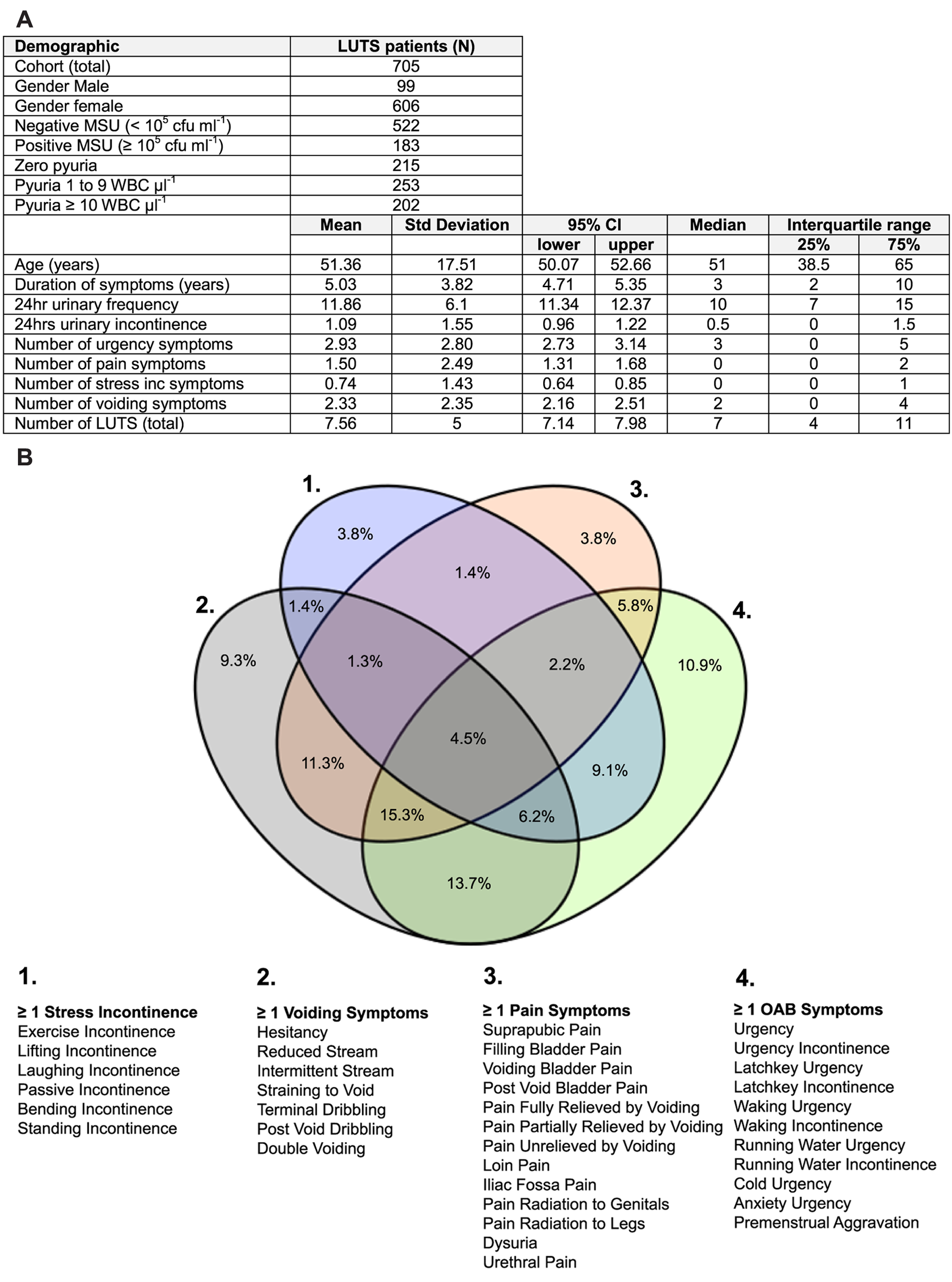

Supplement: Figure S1 — Demographics and symptoms. (A) Key demographic information. (B) A four-way Venn diagram illustrating the overlap of symptoms amongst the 705 patients studied. The ellipses circumscribe patients who had one or more symptoms in the particular subset. Each ellipse corresponds to a numbered list of specific symptoms. The diagram is not scaled to the size of sets. Abbreviations; MSU (mid-stream urine culture); Pyuria (presence of white blood cells in the urine); WBC (white blood cell); inc (incontinence); LUTS (lower urinary tract symptoms); OAB (overactive bladder). (TIF) [file pone.0083637.s001.tif]
